# Longitudinal Associations Between Sleep Habits and Attention-Deficit Hyperactivity Disorder Symptoms in Japanese Children Attending Regular Classrooms

**DOI:** 10.7759/cureus.103818

**Published:** 2026-02-18

**Authors:** Mami Takesada, Shogo Miyashita, Mitsuru Kikuchi

**Affiliations:** 1 Research Center for Child Mental Development, Kanazawa University, Kanazawa, JPN; 2 Psychiatry and Neurobiology, Kanazawa University, Kanazawa, JPN

**Keywords:** adhd, children, longitudinal study, regular classrooms, sleep habits

## Abstract

Aims: To characterize cross-sectional and one‑year longitudinal associations between sleep disturbance and attention-deficit hyperactivity disorder (ADHD) symptom severity in Japanese children diagnosed with ADHD.

Methods: Forty-five children (six to 13-years-old) with clinically diagnosed ADHD, who were unmedicated and attending regular classrooms, were recruited online. At baseline, parents completed the Conners 3 (Japanese parent form), the Social Responsiveness Scale, Second Edition (SRS-2), and the Japanese Sleep Questionnaire for Elementary Schoolers (JSQ‑ES). Thirty-eight participants completed identical assessments at one‑year follow‑up. Pearson’s correlation coefficients examined relationships between JSQ‑ES total T‑scores and Conners 3 Global Index (CGI) and its six factor scales, cross‑sectionally and in change scores (follow‑up minus baseline). Hierarchical multiple regression analyses assessing which ADHD dimensions were predicted by sleep problems after controlling for demographic and autism spectrum disorder (ASD) variables were conducted.

Results: Cross‑sectionally, JSQ‑ES total T‑scores correlated significantly with CGI (r = 0.376, p = 0.010) and with the inattention (IN), hyperactivity/impulsivity (HY), learning problems (LP), and aggression (AG) subscales (all p < 0.05). Longitudinally, only HY changes correlated with JSQ‑ES change scores (r = 0.394, p = 0.014). Regression models confirmed that sleep problems independently predicted overall ADHD severity and AG at baseline, in addition to predicting HY over time.

Conclusions: Sleep disturbances are closely linked to ADHD symptom severity, particularly longitudinal hyperactive/impulsive behaviors, in children, even those without clinical sleep disorder diagnoses. These findings highlight the importance of assessing sleep quality in all children with ADHD, even without major sleep disorders. To draw definitive conclusions about whether improving sleep hygiene reduces HY, further intervention studies are warranted.

## Introduction

Attention-deficit hyperactivity disorder (ADHD) is a highly prevalent neurodevelopmental disorder characterized by inattention (IN), hyperactivity/impulsivity (HY), affecting approximately 5% of children and adolescents [1]. Recently, the importance of understanding and addressing sleep disturbances in neurodevelopmental disorders to improve developmental outcomes and the overall quality of life has been increasingly highlighted [2]. Approximately 60% of children with ADHD experience sleep disturbances, representing a higher prevalence than that observed in their typically developing peers [3]. These disturbances include later bedtimes, prolonged sleep-onset latency, fragmented sleep, and reduced sleep quality [4,5]. Moreover, sleep restriction has been shown to worsen cognitive and behavioral symptoms in typically developing children [6], suggesting that sleep disturbances and behavioral issues may form a reciprocal cycle in children with ADHD, exacerbating clinical presentations.

Although a substantial number of studies have investigated the association between sleep disturbance and the severity of ADHD symptoms, many have relied on cross-sectional designs [7,8], which are inherently incapable of capturing how the relationship between ADHD and sleep changes over time, as children develop, encounter new environments, and face evolving psychosocial stressors. Recently, a large-scale randomized controlled trial of sleep intervention for children with ADHD exhibiting moderate or severe sleep disturbances demonstrated improvements in ADHD symptoms [9]. These findings strongly suggest a causal link between sleep and clinical symptoms, providing meaningful insights into this relationship. However, few longitudinal studies have investigated elementary school children with ADHD who attend regular classes and have no clinically diagnosed sleep disorders, leaving open the question of how home-based sleep patterns and their changes over time may relate to ADHD symptom trajectories.

It is well known that autism spectrum disorder (ASD) and ADHD frequently co-occur [10]. The biological overlap between these two disorders is supported by both neuroimaging studies [11,12] and genetic research [13]. Children with ASD commonly experience severe sleep problems, such as disrupted sleep-wake patterns, difficulty initiating or maintaining sleep, and early morning awakenings at a high frequency [14]. These sleep problems may further aggravate social and behavioral symptoms [15]. Furthermore, sleep issues have been linked to difficulties in social information processing even in young children without a formal ASD diagnosis [16]. Taken together, these findings underscore the importance of accounting for ASD symptoms when examining the relationship between sleep disturbance and clinical outcomes in children with ADHD. Nevertheless, most prior studies have focused on ADHD or ASD independently, while few have investigated ADHD symptoms and sleep while controlling for ASD-related traits.

In light of these considerations, the present study aimed to examine both the cross-sectional and longitudinal associations between sleep disturbance and the severity of ADHD symptoms in a pediatric ADHD population using a statistical model that accounts for ASD symptoms, age, and sex. We applied a standardized sleep assessment tool, the Japanese Sleep Questionnaire for Elementary Schoolers (JSQ-ES), to evaluate sleep problems [17]. Our participants were elementary school children without any significant intellectual disabilities or conspicuous behavioral issues who attended regular classes and were not affected by medication. Baseline and one-year follow-up assessments were performed. This approach allowed us to investigate how changes in home-based sleep habits were related to HY and emotional regulation in Japanese elementary schoolchildren with ADHD, while considering modifiers such as age, sex, and co-occurring ASD symptoms, thereby providing clinically relevant insights.

## Materials and methods

Participants

The study was conducted at the Department of Psychiatry and Behavioral Science, Kanazawa University Graduate School of Medicine, Japan. Participants were recruited via “LITALICO Hattatsu Navi” (LITALICO Inc., Tokyo, Japan, https://h-navi.jp/), an online platform used by parents, caregivers, and educators involved with children diagnosed with developmental disorders. We invited parents of children aged six to 13 years who had been clinically diagnosed with ADHD to join the study. The following cases were excluded from participation: children taking medications that act on the central nervous system, children currently receiving a diagnosis or treatment for sleep disorders or anxiety disorders, children enrolled in special education classes or special support schools for intellectual disabilities, and cases in which a significant environmental change (e.g., a disaster) potentially affecting the child’s sleep in daily life had occurred. The parents of the remaining eligible children were then informed about the study schedule and procedures, and data were collected in the first year (i.e., first research) and one year later (i.e., second research).

Procedure

Parents were informed regarding the nature and objectives of the research at recruitment and at study initiation. Those who consented completed the following questionnaires: ADHD symptoms were measured using Conners 3 (Japanese version, parent form) [18]; autism spectrum traits were measured using the Social Responsiveness Scale, Second Edition (SRS-2, Japanese version) [19]; and sleep-related status was assessed using the JSQ‑ES [17]. In addition, parents were asked whether the child had experienced any significant environmental changes (e.g., relocation), large-scale disasters (earthquakes, eruptions, fires, floods), incidents of violence or abuse, traffic accidents, or the loss of a parent within the past year, as all were deemed factors that could potentially influence behavior or sleep. Each participating family received a gift card worth approximately JPY 6,000 for their cooperation. All parents agreed to allow their children to participate in the study with full knowledge of the experimental nature of the research. Written informed consent was obtained before the start of the experiment, and the Medical Ethics Committee of Kanazawa University approved the methods and procedures used (No. 113601-1).

Psychological tasks

Conners 3

The Conners 3 questionnaire (Japanese version, parent form) was used to comprehensively evaluate the behavioral characteristics of children with ADHD [18]. This standardized assessment tool identifies developmental challenges, including ADHD and conduct-related issues, based on detailed observational information from parents. The Conners 3 has demonstrated very good reliability and validity, with internal consistency coefficients ranging from 0.77 to 0.97 and two- to four-week test-retest reliability coefficients ranging from 0.71 to 0.98 (inter-rater reliability coefficients: 0.52 to 0.94). In the present study, we used the licensed Japanese parent form and followed the publisher’s standardized administration and scoring procedures. In this study, we focused on the Global Index (GI) and major factor scales related to ADHD (i.e., IN, HY, learning problems (LP), executive functioning (EF), aggression (AG), and peer-family relations (PR)).

SRS-2

The SRS-2 was employed to quantitatively assess autistic traits in the participating children [19]. This scale measures behaviors relevant to social communication and interpersonal responsiveness and provides a broad evaluation of ASD symptom severity. The SRS-2 is a revised edition of the original SRS with updated norms. For Japanese children, psychometric evaluation of the Japanese SRS has reported high internal consistency (Cronbach’s α > 0.95) and parent-teacher agreement (r = 0.78), and SRS total scores correlated with ADI-R total scores (ICC = 0.66), supporting convergent validity [20]. The SRS-2, which is widely used in both clinical and research settings, was completed by the parents.

JSQ-ES

To evaluate children’s sleep habits and potential sleep-related problems, we used the JSQ-ES developed at Osaka University [17]. In the original validation study (community sample n = 4,369; clinical sample n = 100), factor analyses supported a nine-factor structure, internal consistency was good (0.876 in the community group and 0.907 in the clinical group), and a total-score cut-off of 80 was proposed. This questionnaire was designed for elementary school-aged children and offers a multifaceted assessment of sleep quality and potential sleep disorders. It was tailored to the educational environment and lifestyle patterns observed in Japan.

Conners 3 (Japanese version; Multi Health Systems, Inc.) and SRS 2 (Japanese version; WPS) were administered under licenses purchased by our laboratory; no copyrighted item content was reproduced, and only aggregate scores are reported. The JSQ ES is made available for research/clinical use by Osaka University with free download and use upon citation.

Statistics

We examined the correlation between the total T-score of the JSQ-ES, which reflects the overall sleep problems, and the Conners 3 Global Index (CGI) T-score, an indicator of overall ADHD symptom severity, as well as the six major factor scales related to ADHD (i.e., IN, HY, LP, EF, AG, and PR) in 45 children with ADHD who participated in the baseline (first-year) evaluation. Pearson’s correlation coefficient was used to assess this relationship.

For the 38 children who also completed the follow-up conducted one year after the initial research, we calculated the change scores (second research − first research) for both the Conners 3 scores and the JSQ-ES total T-score and examined their correlation using Pearson’s correlation coefficient.

As a supplementary analysis, to examine which symptoms of ADHD were related to sleep problems, taking into account the effects of sex, age, and SRS score, we conducted multiple regression analyses using the Conners 3 CGI T-score, as well as each of the six primary factor scales as dependent variables and the JSQ-ES total T-score, sex, age, and SRS score (an index of ASD symptoms) as independent variables. This regression analysis was applied to both the baseline cross-sectional data and longitudinal data derived from the change in scores (second research − first research) at the second-year assessment.

In addition, to provide descriptive information on the overlap between ADHD symptom domains and autistic traits (and to contextualize the inclusion of SRS scores as a covariate), we conducted an exploratory Pearson’s correlation analysis between the SRS-2 total score and the CGI and six primary factor scales at baseline. This targeted analysis was intended to enrich interpretation. All analyses were performed at a significance level (α) of 0.05.

## Results

**Figure 1:**
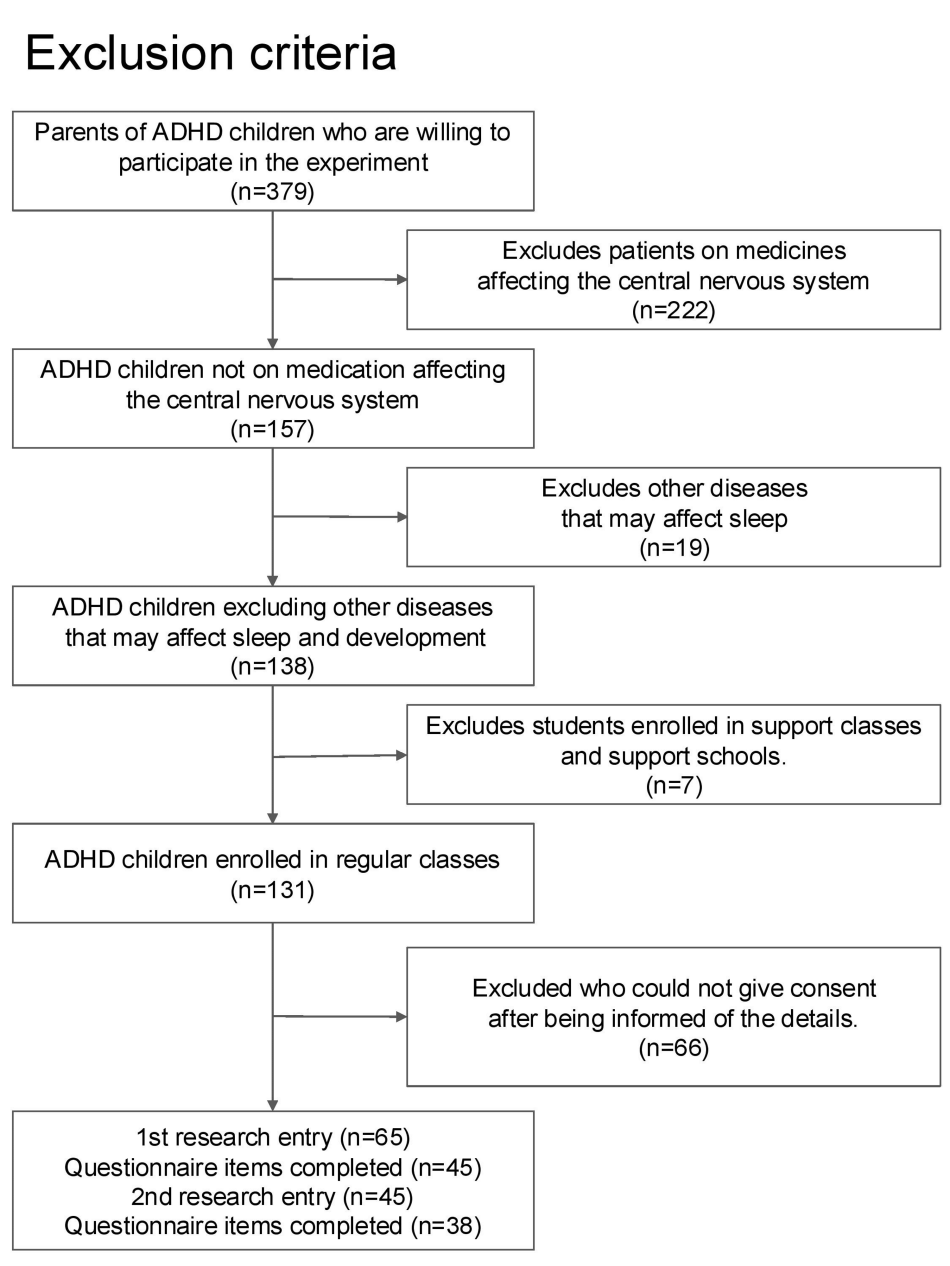
Flow diagram illustrating participant recruitment, screening, and retention across the study period Of 379 families with a clinically diagnosed child with ADHD who initially expressed interest, multiple exclusions were applied based on the use of central nervous system–active medications, concurrent sleep-related clinical diagnoses or treatments, and enrollment in special education. After these exclusions, 65 families participated in the baseline survey; among them, 45 families provided fully completed questionnaire data and were included in the first-year cohort. All 45 families were invited to the follow-up survey, and 38 families ultimately completed the follow-up with fully completed questionnaires, constituting the second-year cohort. ADHD: attention-deficit hyperactivity disorder

As shown in Figure 1, 379 parents of children aged six to 13 years diagnosed with ADHD by a clinician initially expressed interest in the study. From these, we excluded 222 cases in which the child was already taking medication acting on the central nervous system for sleep or developmental issues, 19 cases in which the child was receiving a clinical diagnosis or treatment for sleep disorders or anxiety, and seven cases in which the child was enrolled in a special education class or special support school due to intellectual disabilities. After these exclusions, 65 families participated in the baseline survey; among them, 45 families provided fully completed questionnaire data. Following this screening process, 45 parents agreed to participate in the first year of the study (Table 1). Among these, 18 children were clinically identified by a clinician as having comorbid ASD in addition to ADHD. All 45 families were invited to the follow-up survey, and 38 families ultimately completed the follow-up with fully completed questionnaires, constituting the second-year cohort (i.e., longitudinal follow-up study). Hence, the final sample included 38 children diagnosed with ADHD who met all of the inclusion and exclusion criteria (Table 1).

**Table 1:** Demographic characteristics of all participants SD: standard deviation; SRS-2: Social Responsiveness Scale, Second Edition; JSQ-ES: Japanese Sleep Questionnaire for Elementary Schoolers

	1^st^ research, Mean N = 45	SD	2^nd^ research, Mean N = 38	SD
Gender (male/female)	35/10		31/7	
Chronological age (year)	9.07	1.84	10.13	1.77
Conners 3 (T-score)	73.67	13.61	71.34	10.37
SRS-2 (T-score)	69.76	11.79	67.94	11.67
JSQ-ES (T-score)	67.05	12.23	64.44	12.02

Cross-sectional correlation analysis (first research)

Correlation analysis at the initial assessment (n = 45) revealed a significant positive association between the Conners 3 Global Index T-score (CGI-T), which reflects overall ADHD severity, and the total T-score of the JSQ-ES, which is an index of general sleep problems (r = 0.376, p = 0.010). Further examination of the Conners 3 major factor scales showed significant positive correlations with the total T-score of the JSQ-ES for the four Conners 3 major factor scales (IN scale: r = 0.328, p = 0.027; HY scale: r = 0.350, p = 0.018; LP scale: r = 0.324, p = 0.030; AG scale: r = 0.446, p = 0.002). No other major factor scales exhibited any significant correlations with the JSQ-ES total T-scores. In addition, we examined cross-sectional associations between autistic traits and ADHD symptom domains at baseline (n = 45). The SRS-2 total score showed significant positive correlations with the CGI-T (r = 0.472, p = 0.001), IN (r = 0.321, p = 0.031), HY (r = 0.435, p = 0.003), AG (r = 0.313, p = 0.036), and PR (r = 0.593, p < 0.001). No significant associations were observed with LP (r = 0.156, p = 0.300) or EF (r = 0.226, p = 0.135).

Longitudinal correlation analysis of score changes (second research - first research)

Correlation analysis of score changes (second research - first research) revealed no significant association between changes in the Conners 3 CGI-T score and changes in the JSQ-ES total T-score (r = 0.217, p > 0.050). Among the six Conners 3 major factor scales, only the HY scale showed a significant correlation with changes in the JSQ-ES total T-score (r = 0.394, p = 0.014). No significant correlations were found for the other subscales.

Hierarchical multiple regression

In the cross-sectional (baseline) hierarchical regression analysis, the overall model achieved a good fit for five of the seven dependent variables (GI, IN, HY, AG, and PR). Within these five outcomes, the JSQ-ES total T-score (independent variable) revealed a significant relationship with GI and AG (Table 2).

**Table 2:** Hierarchical multiple regression analysis in cross-sectional research * p<0.05, **p<0.01, ***p<0.005 β: standardized beta coefficient; GI: global index; AG: aggression; SRS-2: Social Responsiveness Scale, Second Edition; JSQ-ES: Japanese Sleep Questionnaire for Elementary Schoolers

Conners GI (N = 45)									
	β in Step1	p-value	β in Step2	p-value	β in Step3	p-value	β in Step4	p-value	t in Step4
Gender	-0.098	0.522	-0.076	0.634	-0.179	0.213	-0.15	0.279	-1.097
Age	-	-	-0.079	0.623	0.214	0.186	0.155	0.326	0.994
SRS-2	-	-	-	-	0.577	0.001***	0.502	0.002***	3.309
JSQ-ES	-	-	-	-	-	-	0.277	0.043*	2.089
F	0.417	0.522	0.328	0.723	5.008	0.005***	5.156	0.002***	-
R	0.098	-	0.124	-	0.518	-	0.583	-	-
Adjusted R^2^	-	-	-	-	0.215	-	0.274	-	-
Conners AG ( N = 45 )									
	β in Step1	p-value	β in Step2	p-value	β in Step3	p-value	β in Step4	p-value	t in Step4
Gender	-0.116	0.449	-0.095	0.553	-0.161	0.305	-0.12	0.410	-0.832
Age	-	-	-0.075	0.638	0.113	0.520	0.029	0.863	0.174
SRS-2	-	-	-	-	0.371	0.033*	0.264	0.106	1.654
JSQ-ES	-	-	-	-	-	-	0.395	0.007**	2.832
F	0.583	0.449	0.399	0.674	1.92	0.141	3.692	0.012*	-
R	0.116	-	0.137	-	0.351	-	0.519	-	-
Adjusted R^2^	-	-	-	-	0.059	-	0.197	-	-

In the longitudinal analysis using score changes, the overall model showed good fit for three of the seven dependent variables (GI, HY, and PR). Of these, only the HY score was significantly associated with the JSQ-ES total T-score (independent variable) (Table 3).

**Table 3:** Hierarchical multiple regression analysis in longitudinal research * p<0.05, **p<0.01, ***p<0.005 β: standardized beta coefficient; HY: hyperactivity/impulsivity; SRS-2: Social Responsiveness Scale, Second Edition; JSQ-ES: Japanese Sleep Questionnaire for Elementary Schoolers

Conners HY ( N = 38 )									
	β in Step1	p-value	β in Step2	p-value	β in Step3	p-value	β in Step4	p-value	t in Step4
Gender	-0.143	0.391	-0.027	0.872	-0.149	0.367	-0.082	0.599	-0.531
Age	-	-	-0.371	0.030	-0.313	0.053	-0.283	0.063	-1.928
SRS-2	-	-	-	-	0.371	0.022*	0.362	0.018*	2.499
JSQ-ES	-	-	-	-	-	-	0.342	0.021*	2.427
F	0.754	0.391	2.965	0.065	4.157	0.013*	5.039	0.003***	-
R	0.143	-	0.381	-	0.518	-	0.615	-	-
Adjusted R^2^	-	-	0.096	-	0.204	-	0.304	-	-

## Discussion

The data from our baseline assessment indicate that sleep-related indices are associated with ADHD characteristics. Moreover, the change-score analysis (i.e., differences between baseline and follow-up) showed that HY was associated with sleep-related factors. Although the present study employed correlational analyses and therefore cannot establish causal relationships, it provides valuable longitudinal data that may help to better understand the temporal associations between sleep and ADHD symptomatology in children. Prior research has indicated that approximately 73% of children with ADHD experience mild to severe sleep problems, with “difficulty initiating sleep” being cited most frequently [7]. Furthermore, children with ADHD show increased nocturnal awakenings as early as age five [21], along with reports of delayed sleep phases and elevated nighttime activity [22,23].

It is well established that adequate sleep is critical to child development, as sleep is deeply involved in neurological, cognitive, emotional, and physical growth. During childhood, nine to 11 hours of sleep per night is recommended [24]. The growth hormone, secreted during non-rapid eye movement (REM) sleep, is believed to support brain development and neuronal repair [25]. Children with consistent sleep routines exhibit higher rates of eye contact and better social development [16]. Hence, sufficient and high-quality sleep is a vital factor for all children, not only those with ADHD. Moreover, many of the detrimental effects of sleep deprivation on cognition, attention, EF, and behavior closely resemble ADHD symptoms [26,27]. Both typically developing children and those with ADHD exhibit increased distractibility when sleep-deprived, and ADHD-specific executive attention may be particularly vulnerable to insufficient sleep [28]. Interestingly, improving sleep has been shown to mitigate behavioral issues among children with ADHD [9], a finding that is consistent with the results of the present study.

Exploratory analyses also demonstrated that autistic traits, as indexed by the SRS-2 total score, were significantly associated with overall ADHD severity and several ADHD symptom domains at baseline, particularly peer/family relations and HY. The strong association with relational functioning is conceptually consistent with the social-communication difficulties characteristic of ASD. Importantly, because our primary sleep-ADHD analyses statistically controlled for SRS-2 scores, the observed longitudinal association between sleep changes and HY cannot be explained solely by overlapping ASD-related traits. These findings underscore the partial but not complete overlap between ASD traits and ADHD symptom domains.

Mechanisms by which sleep deprivation affects the brain and behavior

Neuroimaging studies have shown that sleep deprivation exerts profound effects on brain function and development in multiple ways. Sleep deprivation causes adenosine accumulation in the reward system, leading to its internalization and reduced affinity for dopamine D2/3 receptors, resulting in relative D1 receptor dominance [29]. This receptor imbalance is believed to increase the high-risk/high-return preferences by modulating activation in the nucleus accumbens and insula, brain regions associated with risky decision-making and emotional processing [30]. Whether functional brain features in ASD and ADHD are particularly susceptible to sleep deprivation remains to be fully elucidated; however, further research is warranted.

Limitations

This study has certain limitations. Firstly, our sample size, 45 at baseline and 38 for change-score analyses, may be insufficient to detect certain effects, leading to a potential underestimation of significant findings. More extensive and comprehensive research with a larger sample size is required to draw definitive conclusions. In addition, as this study was observational in design, causal relationships between sleep-related factors and ADHD symptom severity cannot be determined. Further longitudinal and interventional studies are warranted to clarify whether improvements in sleep quality may influence ADHD-related behaviors.

## Conclusions

This study, to our knowledge, is the first longitudinal investigation to evaluate how changes in home-based sleep habits are associated with HY and emotional regulation in Japanese children with ADHD, while considering modifiers such as age, sex, and co-occurring ASD symptoms. The participants were elementary school children with ADHD who had no intellectual disabilities or conspicuous behavioral problems, attended regular classes, and were not receiving medication. Our findings indicate that sleep disturbances are closely linked to ADHD symptom severity, particularly longitudinal hyperactive and impulsive behaviors. These results highlight the importance of assessing sleep quality in all children with ADHD, regardless of the presence of major sleep disorders. Further interventional studies are warranted to clarify whether improving sleep hygiene can reduce HY.
